# Practice Level and Associated Factors Towards the Preventive Measures of COVID-19 Among the General Population; A Systematic Review and Meta-Analysis

**DOI:** 10.3389/fpubh.2022.844692

**Published:** 2022-06-15

**Authors:** Abay Woday Tadesse, Setognal Birara Aychiluhm, Kusse Urmale Mare, Setegn Mihret Tarekegn, Gebeyaw Biset

**Affiliations:** ^1^College of Medicine and Health Sciences, Samara University, Samara, Ethiopia; ^2^Dream Science and Technology College, Dessie, Ethiopia; ^3^Department of Malaria and NTD, Armauer Hansen Research Institute, Addis Ababa, Ethiopia; ^4^College of Medicine and Health Sciences, Wollo University, Dessie, Ethiopia

**Keywords:** systematic review, meta-analysis, preventive practice, COVID-19, population

## Abstract

**Background:**

Studies conducted on the practice of COVID-19 preventive methods across the world are highly inconsistent and inconclusive. Hence, this study intended to estimate the pooled preventive practice and its determinants among the general population.

**Methods:**

This study was conducted using online databases (PubMed, HINARI, Scopus, EMBASE, Science Direct, and Cochrane library database), African Journals online, Google Scholar, open gray and online repository accessed studies. The quality of the included studies was assessed using Newcastle-Ottawa Quality Assessment Scale (NOS). STATA 14.0 software for analysis. The existence of heterogeneity between studies was checked using Cochran Q test and I2 test statistics and then, the presence of publication bias was detected using both funnel plot and Egger's test.

**Results:**

51 studies were included and the pooled level of practice toward the preventive measures of COVID-19 was 74.4% (95% CI: 70.2–78.6%, *I*2 = 99.7%, *P* < 0.001] using a random effects model. Being female [OR = 1.97: 95% CI 1.75, 2.23; *I*2 = 0.0%, *P* < 0.698], rural residence [OR = 0.53: 95% CI 0.44, 0.65; *I*2 = 73.5%, *P* < 0.013], attending higher education level [OR = 1.47: 95% CI 1.18, 1.83; *I*2 = 75.4%, *P* < 0.001], being employed [OR = 2.12: 95% CI 1.44, 3.12; *I*2 = 91.8%, *P* < 0.001], age < 30 [OR = 0.73: 95% CI 0.60, 0.89; *I*2 = 73.9%, *P* < 0.001], and knowledgeable [OR = 1.22: 95% CI 1.09, 1.36; *I*2 = 47.3%, *P* < 0.077] were the independent predictors of adequate practice level.

**Conclusions:**

nearly three-fourths of the general population has an adequate preventive practice level toward COVID-19. Thus, the global, regional, national, and local governments need to establish policies and strategies to address the identified factors.

## Background

Severe acute respiratory infection (SARS) is a group of respiratory tract infections caused by a beta coronavirus (SARS-COV2) (1–3). Corona Virus Disease-2019 (“COVID-19”) is a family of SARS caused by Novel Coronavirus and was first detected in December 2019 in Wuhan, China. Since, the World Health Organization (WHO) has declared it as a pandemic; the virus has been distributed rapidly across the world and it causes high mortality and morbidity (2–5). Globally, there is an estimated 32. million cases and nearly a million (991, 705) deaths at the end of September 2020 (6). As a result, nations across the globe have taken different preventive measures. These include movement restrictions, mask-wearing, hand washing, confinement at home, closure of schools, and other social services (7–9). Hence, appropriate knowledge, attitudes, and practices on preventive measures are mandatory to halt the spread of the COVID-19 outbreak in countries (10, 11). However, studies revealed that the communities have shown still poor knowledge and negative attitude toward the preventive measures of COVID pandemic (12, 13). Besides, studies conducted across the globe have been investigating the knowledge, attitude, and practices on preventive measures of COVID-19 pandemic predominantly focused on health care workers and patients (14–16). However, the studies conducted to date were highly variable and inconsistent to generate evidence regarding the determinants of preventive practice of the general population toward COVID-19. Therefore, this study intended to determine the pooled practice level and its determinants toward the preventive measures of COVID-19 among the general population.

## Methods and Materials

We have used the Preferred Reporting Items for Systematic review and Meta-analyses (PRISMA-2009) (17) to screen the included studies and PRISMA-P 2009 statements to report the findings (Additional File 1) (18).

### Search Strategy

Different online databases (PubMed, HINARI, Scopus, EMBASE, Science direct, Cochrane library database, and African Journals online), Google Scholar, other open gray literatures, and online university repositories were retrieved to include articles conducted on preventive practice toward COVID-19. We have developed different Boolean operators to have comprehensive datasets on preventive practice toward COVID-19. Search MeSH terms: Wuhan coronavirus” OR “COVID-19” OR “novel coronavirus” OR “2019-nCoV” OR “coronavirus disease” OR “SARS-CoV-2” OR “SARS2” OR “severe acute respiratory syndrome coronavirus 2” AND “preventive practice” OR “practice” AND “associated factors” OR “risk factors” OR “determinants”) [Additional file 2]. This study involved studies conducted across the globe to assess the level of practice toward preventive measures of COVID-19 among the adult population.

### Eligibility Criteria to Include Studies

In this systematic review, all observational studies (i.e., cross-sectional, case-control, and cohort), studies reported the level of preventive practice toward COVID-19 and its determinants, and studies published in English language were eligible for this study. Besides, it also included all studies involving the adult population without restriction on the year of publication. However, we excluded studies other than observational studies (case reports, conference reports, and expert opinions) and the studies did not undergo a peer review process.

### Outcome Measurement

This study had two main outcomes. The first outcome was to determine the pooled level of practice toward preventive measures of COVID-19 among the general population. In this study, the adequate practice level was measured by including studies that were correctly classified the level of practice using the median (50%) score or above. Then, the pooled estimate of preventive practice was calculated by dividing the number of population with adequate practice by the total sample size multiplied by 100. The second outcome of the study was to identify determinants of preventive practice using the pooled odds ratio with the corresponding 95% confidence interval.

*Note:* in this study, the general population is defined as all population other than health care professionals who are assumed to have better knowledge and practice toward COVID-19 compared to the general population.

### Data Extraction and Quality Assessment

We had collected the findings of all online databases and eligible articles and it was exported to Microsoft Excel 2016 spreadsheet. Two authors (AWT and SB) extracted the data and reviewed all screened articles. The quality of the included articles was assessed using the Newcastle Ottawa Quality Assessment Scale (NOS) for observational studies (i.e., cross-sectional, case-control, and cohort) was employed (19, 20). The studies with NOS scores of six or more were considered a “good” quality study (low risk) while studies scored less than six were considered as “poor” quality study (high risk) (20) [Additional File 3]. However, all retrieved articles had a score of six or more NOS scores.

### Data Analysis

The extracted data were entered into a Microsoft Excel Database and then it was imported into STATA version 14.0 software with meta-analysis package for further analysis. We had performed a narrative description of the study population, the studies included, the risk factors identified, and the determinants of preventive practice toward COVID-19. The pooled estimate of the level of preventive practice toward COVID-19 was calculated using the random-effects models (21) at 95% confidence intervals. Moreover, the pooled odds ratios were determined with the corresponding 95% confidence intervals for its determinants.

### Heterogeneity and Publication Bias

The Cochran's-Q statistic and *I*^2^ statistic tests with the corresponding *p*-values (22) were used to determine the existence of heterogeneity between studies. In this study, a value of *I*^2^ 25, 50, and 75% were used to declare the heterogeneity test as low, moderate, and high heterogeneity, respectively (22). As a result, we had conducted subgroup analyses, meta-regression, and sensitivity analysis to handle the heterogeneity.

Publication bias was examined by visual inspection of funnel plots (23) and Egger's test (24). Hence, a *p*-value of < 0.05 was considered indicative of statistically significant publication bias.

## Results

### Description of the Included Studies

**Figure 1:**
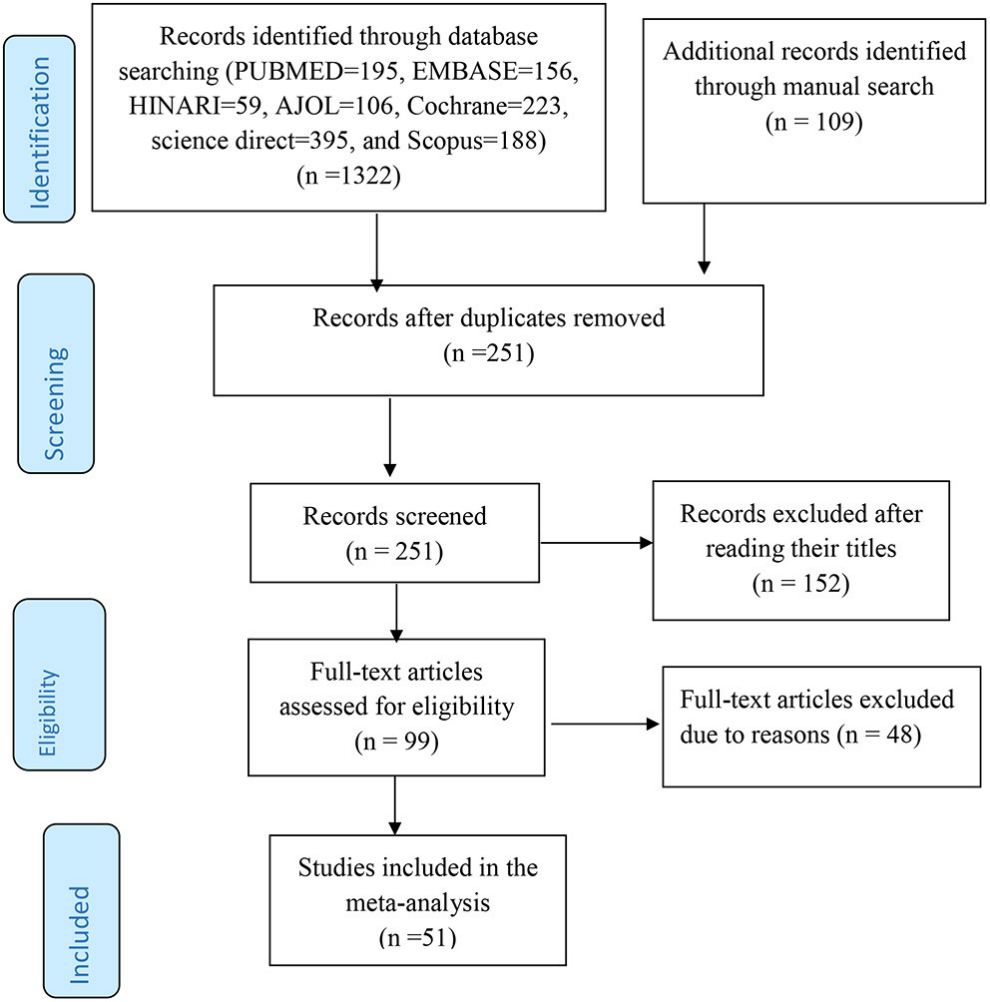
The preferred reporting items for systematic reviews and meta-analyses (PRISMA) flow chart to screen the studies to be included in the review.

In this review, 1,431 studies were retrieved from international databases, African Journals, online, Google Scholar, open gray, and online repositories. The accessed articles were focused on the level of practice toward the preventive measures of COVID-19 and its determinants among the general population. Furthermore, extended references were reached from the published articles. All of he retrieved articles were exported into endnote X8 reference managers and 1,180 articles were removed due to duplication and 152 articles were excluded after review of their titles and abstracts. Therefore, 99 full-text articles were assessed for eligibility and 48 articles were also excluded due to different reasons (i.e., abstracts, case-reports, conference reports, language, and experimental studies). Finally, 51 studies were met the inclusion criteria to undergo the final systematic review and meta-analysis (Figure 1). In this review study, 88,255 study participants were included from 51 observational studies conducted across the world.

### The Pooled Level of Practice Toward the COVID-19 Preventive Measures

The level of practice toward the preventive measures of COVID-19 varies from country to country. In this study, 51 observational studies conducted across the world were included to estimate the level of practice toward preventive measures of COVID-19 among the general population (13, 25–74). Thus, the overall pooled level of adequate practice level toward the preventive measures of COVID-19 was 74.4% (95% CI: 70.2–78.6%, *I*^2^ = 99.7%, *P* < 0.001) using a random effects model [Additional File 4].

### Subgroup Analysis

Different techniques were applied to handle the high level of heterogeneity between the included studies. These include using random effects model, subgroup analysis, meta-regression, and sensitivity analysis.

In this study, subgroup analysis was done based on the region category (i.e., low-income, middle-income, and high-income) of the countries where the included studies were conducted and sample size category (i.e., sample size <380 and sample size ≥380). As a result, the pooled level of practice toward the preventive measures of COVID-19 in low-income, middle-income, and high-income countries was 69.0% [95% CI 62–76: *I*^2^ = 99.1%, *p* < 0.001), 81.0% (95% CI 75.0–87.0: *I*^2^ = 99.4%, *p* < 0.001), and 78.0% (95% CI 70–86: *I*^2^ = 99.8%, *p* < 0.001) respectively [Additional File 5]. Regarding sample size, the pooled level of practice on the preventive measures of COVID-19 was 81.0% (95%CI 75.1–86.0: *I*^2^ =75.5%, *P* < 0.01) and 74.0% (95%CI 70.0–78.0: *I*^2^ = 99.7%, *P* < 0.001) among studies involving fewer than 380 and 380 or more study participants, respectively [Additional File 6].

### Publication Bias

**Figure 2:**
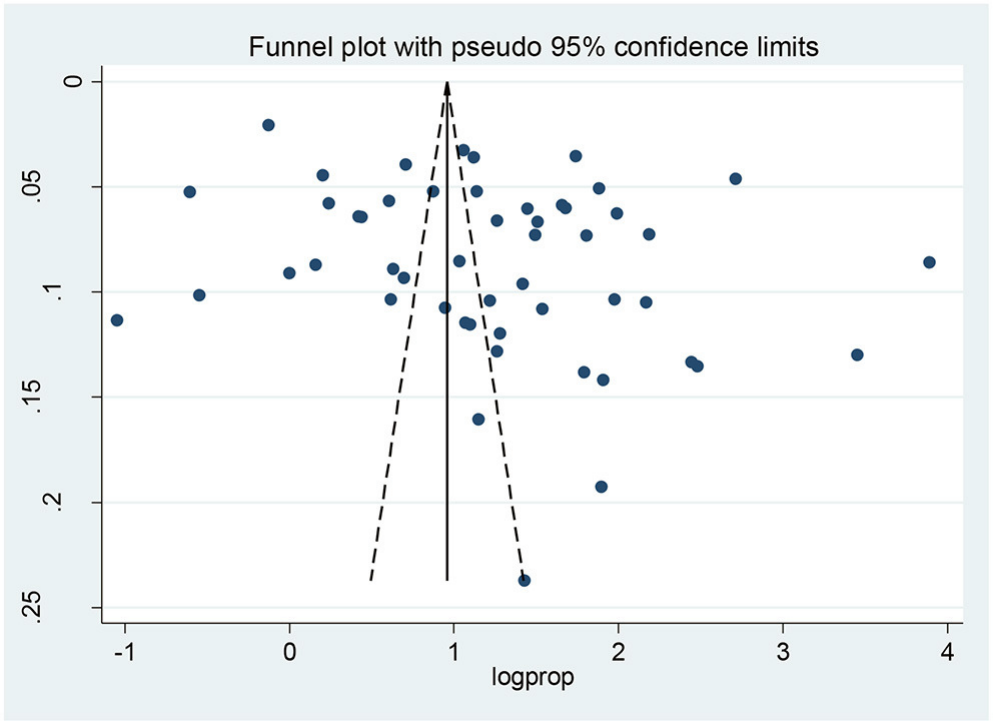
Funnel plot to determine the presence of publication bias between 51 included studies.

To identify the presence of publication bias, both a funnel plot and Egger's test were performed. Visual inspection of the funnel plot showed an asymmetrical distribution, which indicated the presence of publication bias (Figure 2). The finding of publication bias was confirmed following the Egger's test (*p* <0.013).

### Trim and Fill Analysis

**Figure 3:**
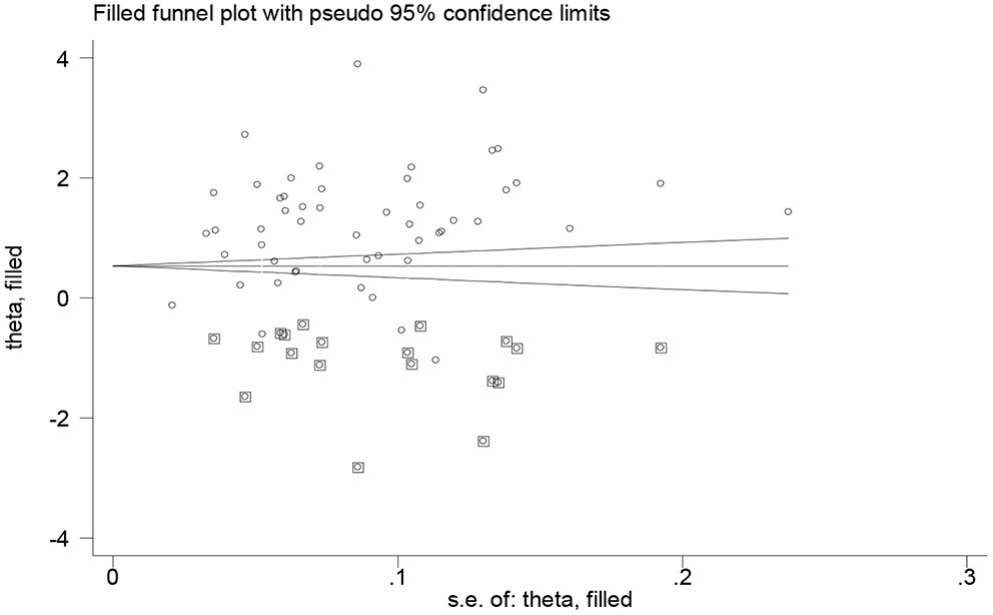
The trim and fill analysis to hand out the publication bias of the included studies.

In this review, the authors confirmed that the presence of significant publication bias that may be subjected to unpublished small studies. Thus, to handle this problem, the authors did trim and fill analysis and 19 studies were filled (Figure 3).

### Sensitivity Analysis

We conducted a sensitivity analysis to assess the effect of any individual study on the pooled effect size. However, the sensitivity analysis done using a random effects model revealed that no single study affected the overall level of practice with the preventive measures of COVID-19 (Additional File 7).

### Meta-Regression Analysis

To investigate the possible source(s) of variation across the included studies, we performed meta-regression analysis using region (high, middle, and low-income), sample size, and quality of the score as covariates of interest. Thus, the results of this meta-regression analysis showed that region category was significantly associated with the presence of heterogeneity (*p* <0.028) (Table 1).

**Table 1:** Meta regression to identify variables for heterogeneity between studies.

**List of variables**	**Coefficient**	**Std. err**.	***t***	***P* > *t***	**[95% Confidence Interval]**	
					**Lower limit**	**Upper limit**
Region category	−0.0459398	0.020416	−2.25	0.028*	−0.0867253	−0.0051542
Sample size	5.73e-06	0.0000102	0.56	0.576	−0.0000147	0.0000261
Quality score	−0.0373856	0.0192668	−1.94	0.057	−0.0758754	0.0011043
Constant	1.093101	0.1444608	7.57	0	0.8045072	1.381694

### Factors Associated With the Level of Practice Toward COVID-19 Prevention

#### Sex of the Participants

**Figure 4:**
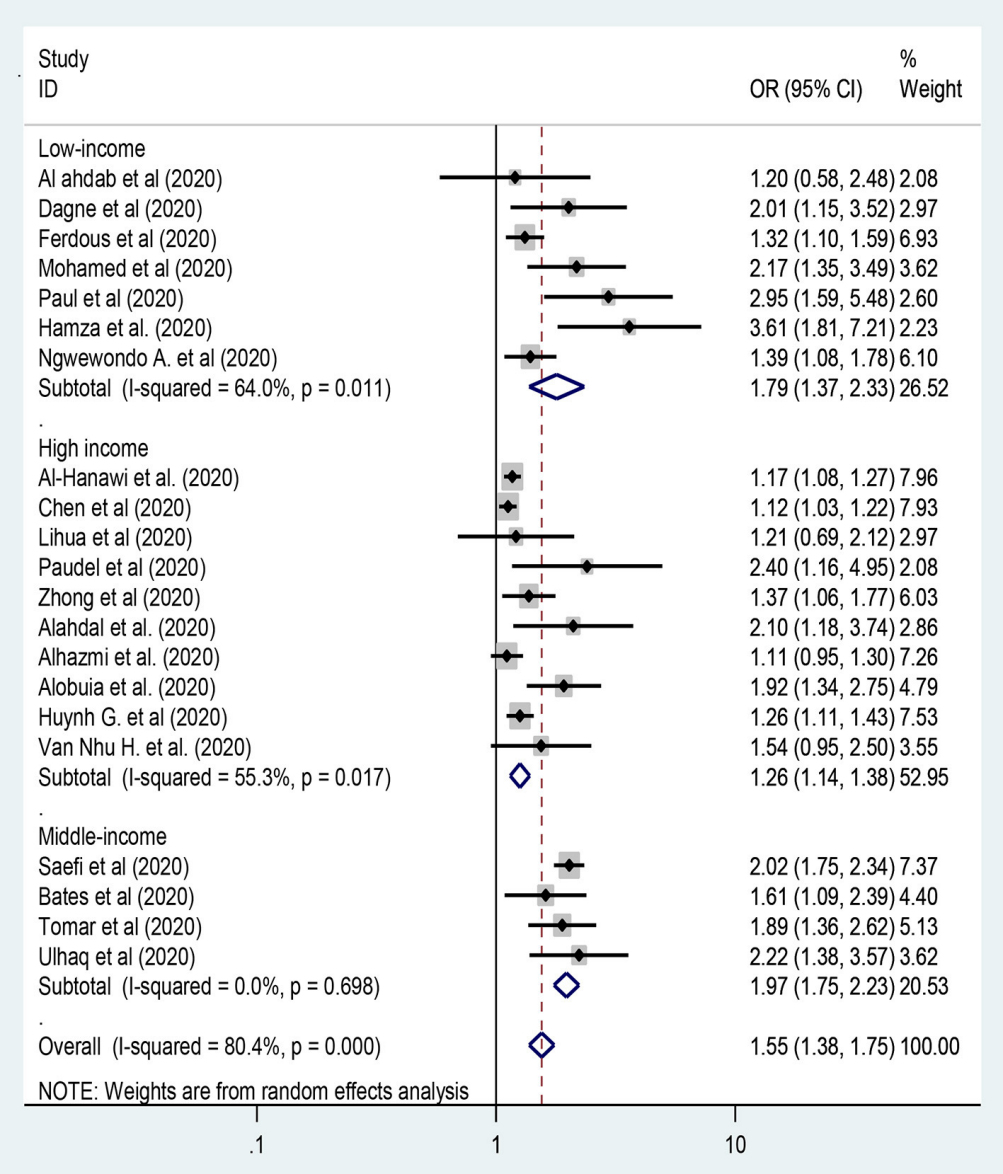
The association between sex of the participants and level of practice toward COVID-19.

In this meta-analysis, twenty-one studies were included to assess the association between sex of the participants and preventive practice toward COVID-19 (13, 27, 28, 33, 34, 37, 39, 41, 42, 45, 50–56, 60, 65, 67, 73). Hence, female participants in middle-income countries were twice more likely to have adequate practice on the preventive measures of COVID-19 [OR = 1.97: 95% CI 1.75, 2.23: *I*^2^ = 0.0%, *P* = 0.698] compared to male participants (Figure 4). In this meta-analysis, there was heterogeneity between the included studies while we applied random-effects model and then subgroup analysis by region was done to handle the variation between studies.

### Knowledge Level as A Factor of Preventive Practice Toward COVID-19

**Figure 5:**
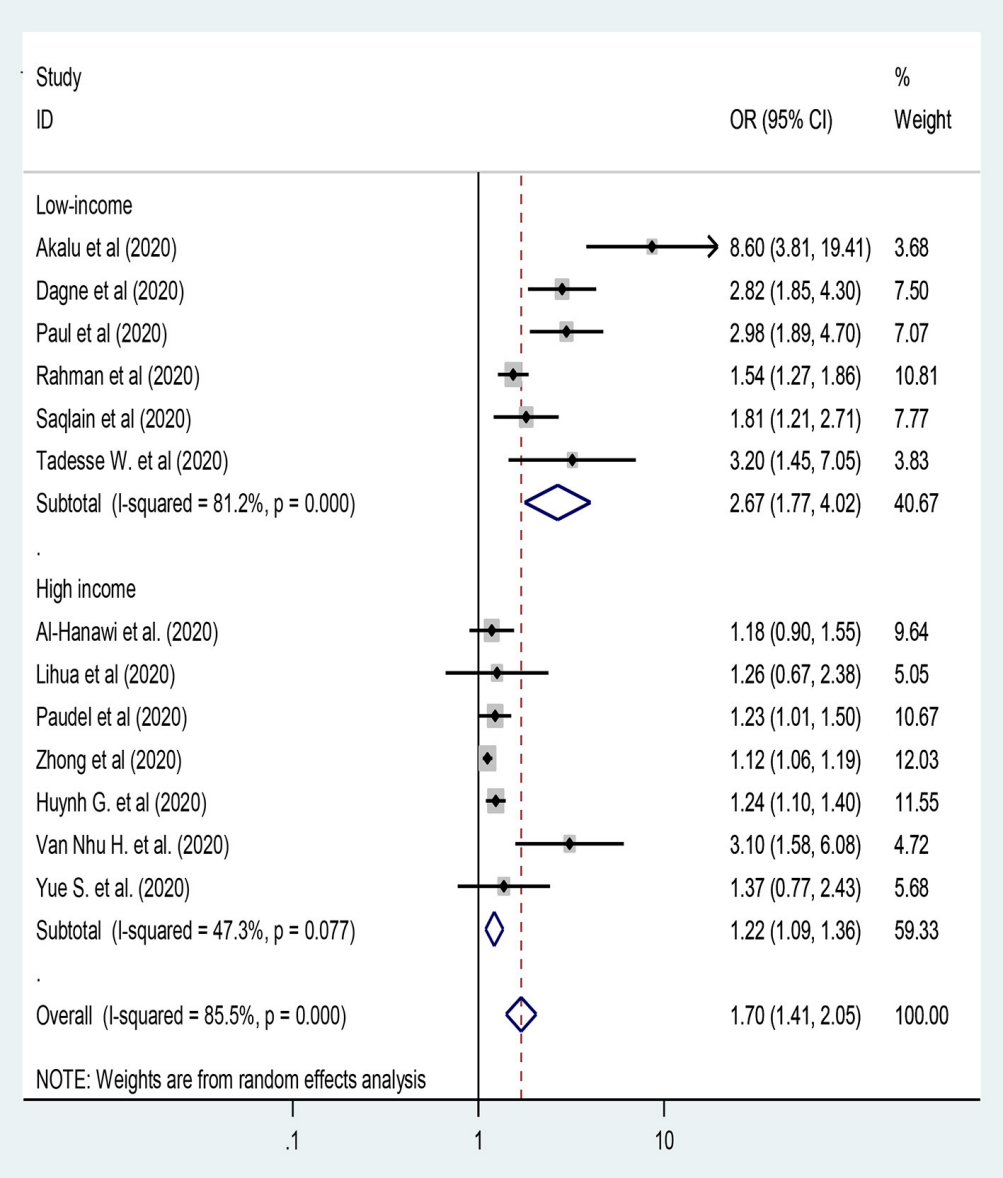
The knowledge level of the participants and practice toward COVID-19.

In this study, thirteen studies were included to assess knowledge level as a predictor of practice of preventive measures toward COVID-19 (26, 28, 34, 37, 41, 42, 44, 46, 49, 53, 65, 73, 74) by using random-effect model analysis. However, still we were unable to handle the heterogeneity between studies [*I*^2^ = 86.2%, *P* < 0.001]. Hence, subgroup analysis was done using country development category [i.e., low-and high-income]. Hence, participants from high-income countries and with adequate knowledge had 22% more likely to have adequate practice toward COVID-19 compared to their counterparts [OR = 1.22: 95% CI 1.09, 1.36; *I*^2^ = 47.3%, *P* = 0.077] (Figure 5).

### Residence Areas and Practice of the COVID-19 Preventive Measures

**Figure 6:**
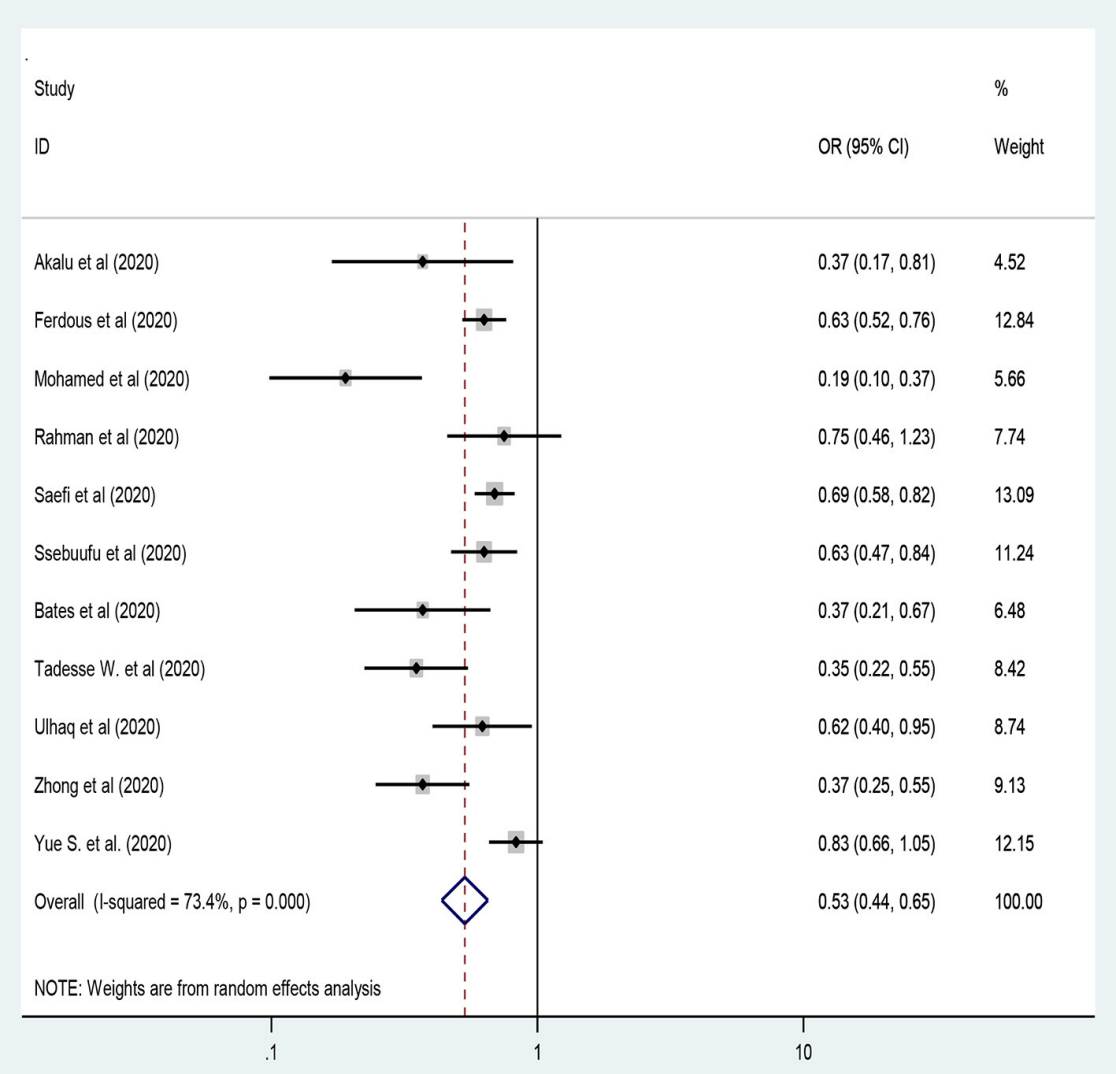
The residence of the participants and preventive practice toward COVID-19.

Eleven studies were included to assess residence in rural areas as a negative predictor of adequate practice of preventive measures toward COVID-19 (13, 26, 39, 44, 45, 48, 49, 51–53, 74) by using a random-effect model that was applied to handle the variation between studies [*I*^2^ = 71.8%, *P* < 0.001]. Hence, people living in rural areas had 47% less likely to have adequate practice on the preventive measures of COVID-19 compared to their counterparts [OR = 0.53: 95% CI 0.44, 0.65; *I*^2^ = 73.5%, *P* < 0.001] [Figure 6].

### Education Level and Practice of the COVID-19 Preventive Measures

**Figure 7:**
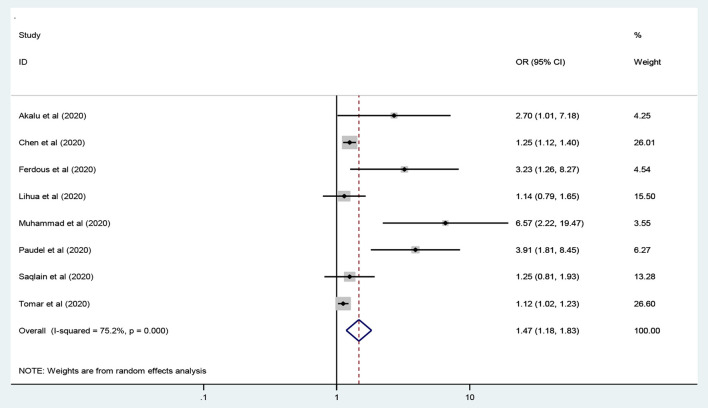
The education level of the participants and preventive practice toward COVID-19.

Seven studies were included to assess education level as an independent predictor of level of practice toward COVID-19 the preventive measures (13, 26, 33, 37, 41, 46, 50). The meta-analysis results revealed that participants who had attended higher education levels were 47% more likely to practice preventive measures toward COVID-19 compared to their counterparts [OR = 1.47: 95% CI 1.18, 1.83: *I*^2^ = 75.4%, *P* < 0.001]. We applied random-effects model to handle the variation between studies (Figure 7).

### Employment Status and Practice of the COVID-19 Preventive Measures

**Figure 8:**
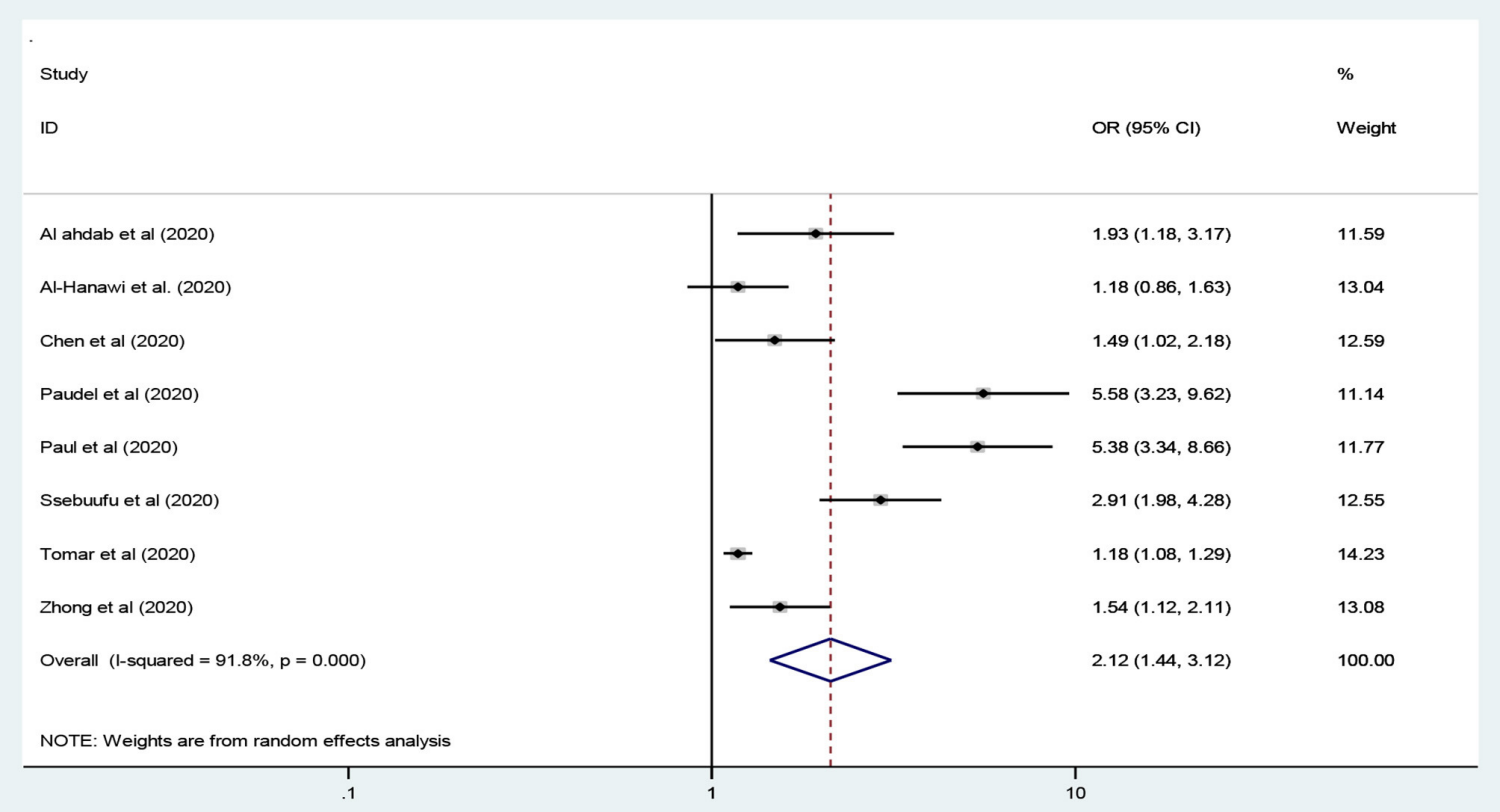
Employment status and preventive practice toward COVID-19.

In this study, eight studies were included to assess being employed as a predictor of adequate practice toward the preventive measures of COVID-19 globe (27, 28, 33, 41, 42, 48, 50, 53). As a result, employed participants had twice higher odds of adequate practice compared to unemployed participants [OR = 2.12: 95% CI 1.44, 3.12, *I*^2^ = 91.2%, *P* < 0.001] using a random-effects model analysis (Figure 8).

### Age of the Participants and Practice of the COVID-19 Preventive Measures

**Figure 9:**
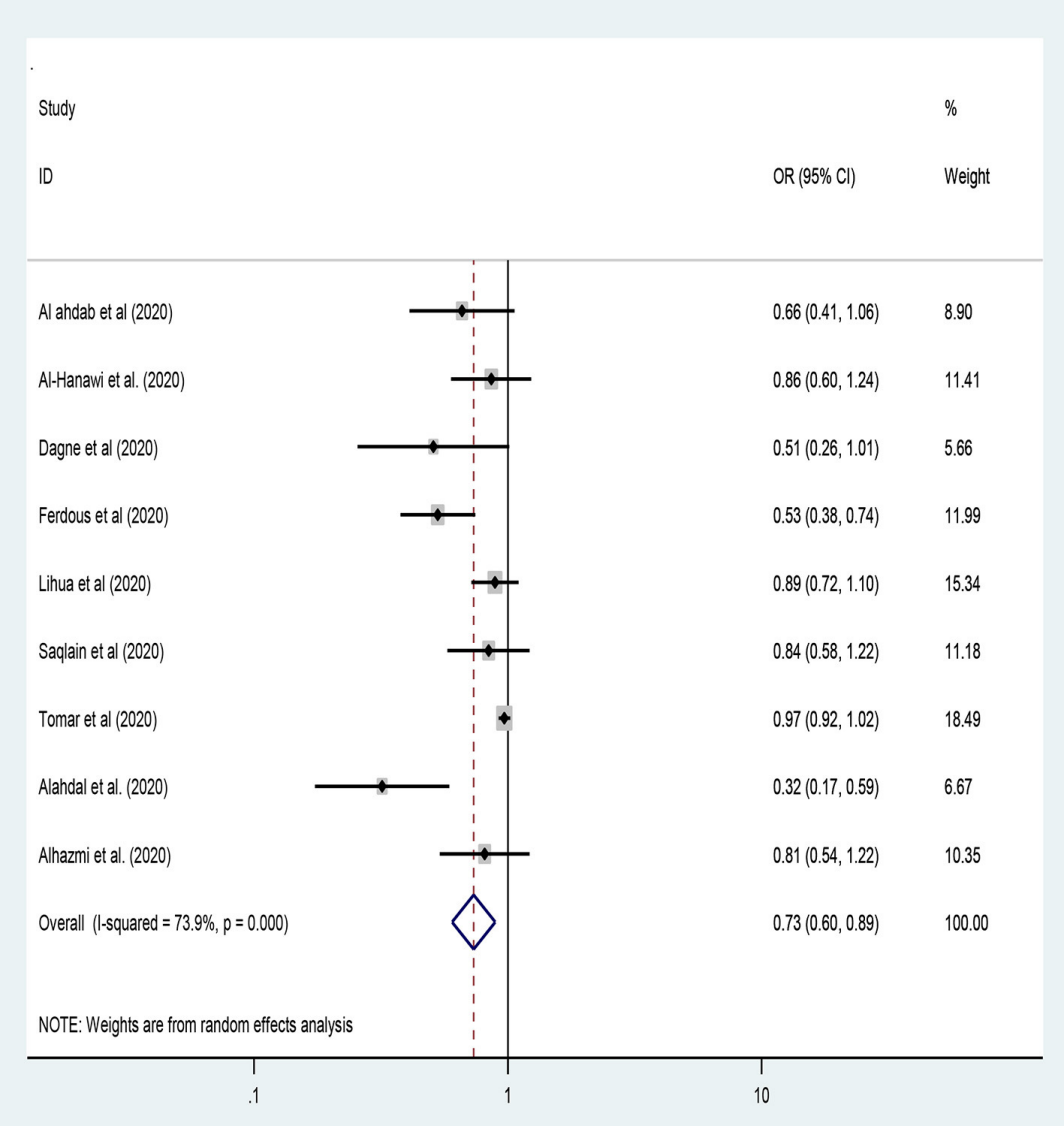
The age category of participants and preventive practice toward COVID-19.

In this study, nine studies were included to assess the association between the age of the participants and the level of practice of preventive measures toward COVID-19 (13, 27, 28, 34, 37, 46, 50, 54, 55). Hence, young age [<30 years of age] participants had 27% lower odds of adequate practice toward the preventive measures of COVID-19 compared to their counterparts [OR = 0.73: 95% CI 0.60, 0.89, *I*^2^ = 73.9%, *P* < 0.001]. Besides, the random-effect model was employed to handle the heterogeneity between studies [Figure 9].

## Discussion

In this systematic review and meta-analysis, 51 observational studies were retrieved across the world focusing on the level of practice toward the preventive measures of COVID-19 and its determinants. In this review, the overall pooled level of adequate practice on the preventive measures of COVID-19 was 74.4% [95% CI: 70.2–78.6%] that was done using a random effects model. This finding is lower than studies conducted Pakistan (80.5%) (46), Sudan (89.9%) (39), Shaanxi Province, China (87.9%) (75), Uganda (85.3%) (48). However, this finding is higher than studies conducted in Ethiopia (26.1 to 72.5%) (26, 34, 49, 76), Bangladesh (55.1%) (13), and Pakistan (57.3%) (15). The differences in the practice of preventive measures could have been subjected to variation in the cut-off values to classify good or poor practices. For instance, most of the previous studies have been used the score of 80% and above to classify adequate practice, while the current study was classified based on the median score (50% or more), to consider studies with a good level of practice toward the preventive measures of COVID-19. In addition, the discrepancies might be due to differences in sample size, in which the current study involved a large sample sizes. Therefore, further investigation should be done to identify the main reasons for this variation across the regions.

On the other hand, the subgroup analysis indicated that the pooled level of practice toward the preventive measures of COVID-19 in low-income, middle-income, and high-income countries was 69, 81, and 78%, respectively. This indicated that substantial heterogeneity of the level of preventive practice across countries. Such differences might be subject to low adherence of preventive practice toward COVID-19 in low-income countries. The other reason for the lower prevalence in these regions might be due to more studies were included in the review process.

This study revealed that females from middle-income countries had twice higher odds of adequate practice on the preventive measures of COVID-19 [OR = 1.97] compared to male participants, which was similar to the findings of studies conducted across the world (13, 27, 28, 33, 34, 37, 39, 41, 42, 45, 50–56, 60, 65, 67, 73). In most of the countries, females are more responsible to take care than the family members. Besides, females are more likely to obey the regulations and rules of the government so that they are more likely to adhere the practice of preventive measures toward COVID-19 compared to males.

In this study, knowledge level was the independent predictor of adequate practice on the preventive measures of COVID-19. Hence, the subgroup analysis showed that participants from high-income countries and with adequate knowledge had 22% more likely to have adequate practice toward COVID-19 [OR = 1.22] compared to their counterparts. Evidences across the world revealed that (26, 28, 34, 37, 41, 42, 44, 46, 49, 53, 73, 74) adequate knowledge level positively correlated with adequate practice scores toward the preventive measures of COVID-19.

The meta-analysis results revealed that participants who had attended higher education levels were 47% more likely to have adequate practice toward the preventive measures COVID-19 compared to their counterparts [OR = 1.47]. Evidences of several studies conducted in different countries 19 (13, 26, 33, 37, 41, 46, 50) supported this finding. When the education level of the participants increases, they will have good knowledge regarding the preventive measures of COVID-19. This in terms improves the practice of the participants on preventive measures upon COVID-19.

In this study, the subgroup analysis by region category pointed out that participants from middle-income countries and those living in rural areas had 47% less likely to have adequate practice toward preventive measures of COVID-19 compared to their counterparts [OR = 0.53]. This finding is similar to the findings from different studies conducted in several countries (13, 26, 39, 44, 45, 48, 49, 51–53, 74). In most countries, people living in rural settings are not accessible to the preventive measures provided by the government so that they are less likely to practice the preventive measures of the COVID-19 compared to people in urban settings.

This study indicated that the odds of adequate practice toward the preventive measures of COVID-19 was twice higher among employed participants compared to unemployed participants [OR= 2.12]. This finding is similar to studies conducted across the globe (27, 28, 33, 41, 42, 48, 50, 53). Employed individuals have the chance to be exposed to preventive measures since the employers safeguard their employees to be free of the impact of the COVID-19 pandemic. Thus, employed people are more likely to have adequate practice on the preventive measures of COVID-19 compared to non-employed individuals.

Worldwide, studies revealed that people under 30 years of age are less likely to be adhered to the preventive practice of COVID-19 (13, 27, 28, 34, 37, 46, 50, 54, 55). In our study, young age (<30 years of age) participants had 27% lower odds of adequate practice toward the preventive measures of COVID-19 compared to their counterparts [OR = 0.73]. In adulthood, people are less likely to adhere to the preventive measures recommended by the government so that they will have less compliance with practice of the COVID-19 preventive measures.

### Limitations of the Study

The first limitation of the study was only English articles or reports were considered to carry out the analysis. Even though the quality of each study was assessed by using The Newcastle-Ottawa Scale, inter-author bias might be occurred with the leveling of the scale of each article. Reviewing of different characteristics of the involved cases with different sampling methods was also the other limitation of this study.

## Conclusions

In this review, nearly three-fourths of the participants worldwide had a pooled levels of adequate practice toward COVID-19. Being female, rural residence, higher education level, being employed, age <30, and above median knowledge score were independent predictors of preventive practice toward COVID-19. Thus, the national and local governments should develop effective and inclusive prevention strategies to address students who are at home due to COVID-19 pandemic.

## Data Availability Statement

The data analyzed in this study is subject to the following licenses/restrictions: we will provide the dataset based on request when necessary. Requests to access these datasets should be directed to abaywonday@su.edu.

## Author Contributions

AWT, SBA, KUM, SMT, and GB have conceived the title, write the significance of the study, generate the research questions, and write the methods section. AWT and SBA have done the data extraction and quality assessment. All authors critically reviewed and approved the final manuscript.

## Conflict of Interest

The authors declare that the research was conducted in the absence of any commercial or financial relationships that could be construed as a potential conflict of interest.

## Publisher's Note

All claims expressed in this article are solely those of the authors and do not necessarily represent those of their affiliated organizations, or those of the publisher, the editors and the reviewers. Any product that may be evaluated in this article, or claim that may be made by its manufacturer, is not guaranteed or endorsed by the publisher.

## Abbreviations

OR: Odds Ratio
CI: Confidence Interval
COVID 19: Corona Virus Diseases 19
FMOH: Federal Ministry of Health
PRISMA-2009: Preferred Reporting Items for Systematic review and Meta-analyses
WHO: World Health Organization.

## References

[1] Skowronski DM Astell C Brunham RC Low DE Petric M . Severe acute respiratory syndrome (SARS): a year in review. Annu Rev Med. (2005) 56:357–81. 10.1146/annurev.med.56.091103.134135 15660517

[2] Wu D Wu T Liu Q Yang Z. The SARS-CoV-2 outbreak: what we know. International Journal of Infectious Diseases. (2020). 10.1016/j.ijid.2020.03.004 32171952 PMC7102543

[3] Zumla A Hui DS Perlman S. Middle East respiratory syndrome. Lancet. (2015) 386:995–1007. 10.1016/S0140-6736(15)60454-8 26049252 PMC4721578

[4] Lu H Stratton CW Tang YW. Outbreak of pneumonia of unknown Etiology in Wuhan China: the mystery and the miracle. J Med Virol. (2020) 92:401–2. 10.1002/jmv.25678 31950516 PMC7166628

[5] *CDC for disease Prevention: people who are at higher risk for severe illness*. Available online at: https://www.cdc.gov/media/releases/2020 (accessed April 5, 2020).

[6] WHO. Coronavirus disease (COVID-19) Situation Report – 201. Available online at: https://www.who.int/emergencies/diseases/novel-coronavirus-2019/situation-reports-131.2020

[7] Burke RM. Active monitoring of persons exposed to patients with confirmed COVID-19—United States, January–February 2020. MMWR Morbidity and mortality weekly report 2020, 69. 10.15585/mmwr.mm6909e1 32134909 PMC7367094

[8] Zettler I Schild C Lilleholt L Böhm R. Individual differences in accepting personal restrictions to fight the COVID-19 pandemic: Results from a Danish adult sample. PsyArXiv [Preprint]. (2020).

[9] Dong Y Mo X Hu Y Qi X Jiang F Jiang Z . Epidemiology of COVID-19 among children in China. Pediatrics. (2020). 10.1542/peds.2020-0702 32179660

[10] Hu W Su L Qiao J Zhu J Zhou Y. Countrywide quarantine only mildly increased anxiety level during COVID-19 outbreak in China. medRxiv [Preprint]. (2020). 10.1101/2020.04.01.20041186

[11] Liu S Yang L Zhang C Xiang Y-T Liu Z Hu S . Online mental health services in China during the COVID-19 outbreak. The Lancet Psychiatry. (2020) 7:e17–8. 10.1016/S2215-0366(20)30077-8 32085841 PMC7129099

[12] Li S Wang Y Xue J Zhao N Zhu T. The impact of COVID-19 epidemic declaration on psychological consequences: a study on active Weibo users. Int J Environ Res Public Health. (2020) 17:2032. 10.3390/ijerph17062032 32204411 PMC7143846

[13] Ferdous MZ Islam MS Sikder MT Mosaddek AS Zegarra-Valdivia JA Gozal D. Knowledge, attitude, and practice regarding COVID-19 outbreak in Bangladesh: An online-based cross-sectional study. PLoS ONE (2020) 15:e0239254. 10.1371/journal.pone.0239254 33035219 PMC7546509

[14] Zhang M Zhou M Tang F Wang Y Nie H Zhang L . Knowledge, attitude, and practice regarding COVID-19 among healthcare workers in Henan, China. J Hospital Infect. (2020) 105:183–7. 10.1016/j.jhin.2020.04.012 32278701 PMC7194961

[15] Muhammad K Saqlain M Hamdard A Naveed M Umer MF Khan S . Knowledge, attitudes, and practices of community pharmacists about COVID-19: a cross-sectional survey in two provinces of Pakistan. medRxiv [Preprint]. (2020). 10.1101/2020.05.22.20108290 PMC8129683 33588970

[16] Olum R Chekwech G Wekha G Nassozi DR Bongomin F. Coronavirus Disease-2019: Knowledge, attitude, and practices of health care workers at Makerere university teaching hospitals, Uganda. Front Pub Health. (2020) 8:181. 10.3389/fpubh.2020.00181 32426320 PMC7204940

[17] Moher D Liberati A Tetzlaff J Altman DG. Preferred reporting items for systematic reviews and meta-analyses: the PRISMA statement. Bmj. (2009) 339:b2535. 10.1136/bmj.b2535 19622551 PMC2714657

[18] Shamseer L Moher D Clarke M Ghersi D Liberati A Petticrew M . Preferred reporting items for systematic review and meta-analysis protocols (PRISMA-P) 2015: elaboration and explanation. BMJ: Br Med J. (2015) 349:g7647. 10.1136/bmj.g7647 25555855

[19] Peterson J Welch V Losos M Tugwell PJ. The Newcastle-Ottawa Scale (NOS) for Assessing the Quality of Nonrandomised Studies in Meta-Analyses. Ottawa, ON: Ottawa Hospital Research Institute (2011). p. 1–2.

[20] McPheeters ML Kripalani S Peterson NB Idowu RT Jerome RN Potter SA . Closing the quality gap: revisiting the state of the science (vol. 3: quality improvement interventions to address health disparities). Evid Rep Technol Access (Full Rep). (2012) 1–475. 24422952 PMC4781280

[21] DerSimonian R Laird N. Meta-analysis of clinical trials. Control Clin Trials. (1986) 7:177–88. 10.1016/0197-2456(86)90046-2 3802833

[22] Higgins JP Thompson SG. Quantifying heterogeneity in a meta-analysis. Stat Med. (2002) 21:1539–58. 10.1002/sim.1186 12111919

[23] Liu JL. The role of the funnel plot in detecting publication and related biases in meta-analysis. Evid Based Dent. (2011) 12:121–2. 10.1038/sj.ebd.6400831 22193659

[24] Egger M Davey Smith G Schneider M Minder C. Bias in meta-analysis is detected by a simple graphical test. Bmj. (1997) 315:629–34. 10.1136/bmj.315.7109.629 9310563 PMC2127453

[25] Adesegun OA Binuyo T Adeyemi O Ehioghae O Rabor DF Amusan O . The COVID-19 crisis in Sub-Saharan Africa: knowledge, attitudes, and practices of the Nigerian public. Am J Trop Med Hyg. (2020) 107:1997. 10.4269/ajtmh.20-0461 32975179 PMC7646756

[26] Akalu Y Ayelign B Molla MD. Knowledge, attitude and practice towards COVID-19 among chronic disease patients at Addis Zemen hospital, Northwest Ethiopia. Infect Drug Resist. (2020) 13:1949–60. 10.2147/IDR.S258736 32612371 PMC7322118

[27] Al Ahdab S. Knowledge, attitudes, and practices (KAP) towards pandemic COVID-19 among Syrians. Res Square [Preprint]. (2020). 10.21203/rs.3.rs-27859/v133546652

[28] Al-Hanawi M Angawi K Alshareef N Qattan A Helmy H Abudawood Y . Knowledge, attitude and practice toward COVID-19 among the public in the Kingdom of Saudi Arabia: a cross-sectional study. Front Public health. (2020) 8:217. 10.3389/fpubh.2020.00217 32574300 PMC7266869

[29] Ali M Uddin Z Banik PC Hegazy FA Zaman S Ambia AS . Knowledge, attitude, practice, and fear of COVID-19: An online-based cross-cultural study. Int J Ment Health Addict. (2021) 1–16. 10.1007/s11469-021-00638-4. [Epub ahead of print]. 34483782 PMC8404540

[30] Anikwe CC Ogah CO Anikwe IH Okorochukwu BC Ikeoha CC. Coronavirus disease 2019: knowledge, attitude, and practice of pregnant women in a tertiary hospital in Abakaliki, southeast Nigeria. Int J Gynecol Obst. (2020) 151:197–202. 10.1002/ijgo.13293 32608513 PMC9087705

[31] Azlan AA Hamzah MR Sern TJ Ayub SH Mohamad E. Public knowledge, attitudes and practices towards COVID-19: a cross-sectional study in Malaysia. PLoS ONE. (2020) 15:e0233668. 10.1371/journal.pone.0233668 32437434 PMC7241824

[32] Byanaku A Ibrahim M. Knowledge, attitudes, and practices (KAP) towards COVID-19: a quick online cross-sectional survey among Tanzanian residents. medRxiv [Preprint]. (2020).

[33] Chen X Ran L Liu Q Hu Q Du X Tan X. Hand hygiene, mask-wearing behaviors and its associated factors during the COVID-19 epidemic: a cross-sectional study among primary school students in Wuhan, China. Int J Environ Res Public Health. (2020) 17:2893. 10.3390/ijerph17082893 32331344 PMC7215913

[34] Dagne H Alemu KA Dagnew B Taddesse D Alemayehu AM Andualem Z . Prevention practice and associated factors of coronavirus disease 2019 (COVID-19) outbreak among educated ethiopians: an online based cross-sectional survey. Res Square [Preprint]. (2020). 10.21203/rs.3.rs-34504/v1

[35] Joshi K Jamadar D. Knowledge, attitude and practices regarding COVID-19 among medical students-A cross sectional study. Int J Adv Community Med. (2020) 3:1–5. 10.33545/comed..v.i.142

[36] Lau LL Hung N Go DJ Ferma J Choi M Dodd W Wei X: Knowledge attitudes attitudes and practices of COVID-19 among income-poor households in the Philippines: a cross-sectional study. J Glob Health. (2020) 10:7. 10.7189/jogh.10.011007 32566169 PMC7294392

[37] Lihua M Ma L Liu H Jiang N Wang S Jiang X. Knowledge, beliefs/attitudes and practices of rural residents in the prevention and control of COVID-19: an online questionnaire survey. Res Square [Preprint]. (2020) 1–12. 10.21203/rs.3.rs-22257/v1 33124537 PMC7695081

[38] Maheshwari S Gupta PK Sinha R Rawat P. Knowledge, attitude, and practice towards coronavirus disease 2019 (COVID-19) among medical students: a cross-sectional study. J Acute Disease. (2020) 9:100. 10.4103/2221-6189.283886

[39] Mohamed A Elhassan E Mohamed AO Mohammed AA Mahgoop MA Sharif ME . Knowledge, attitude and practice of the Sudanese people towards COVID-19: an online survey. Res Square [Preprint]. (2020). 10.21203/rs.3.rs-32375/v1 33535995 PMC7856621

[40] Naser AY Dahmash EZ Alwafi H Alsairafi ZK Al Rajeh AM Alhartani YJ . Knowledge and practices towards COVID-19 during its outbreak: a multinational cross-sectional study. medRxiv [Preprint]. (2020). 10.1101/2020.04.13.20063560

[41] Paudel S Shrestha P Karmacharya I Pathak OK: Knowledge attitude and practices (KAP) towards COVID-19 among Nepalese residents during the COVID-19 outbreak: an online cross-sectional study. Res Square [Preprint]. (2020). 10.21203/rs.3.rs-31044/v1

[42] Paul A Sikdar D Hossain MM Amin MR Deeba F Mahanta J . Knowledge, attitude and practice towards novel corona virus among bangladeshi people: implications for mitigation measures. medRxiv [Preprint]. (2020). 10.1101/2020.05.05.20091181 PMC7467312 32877449

[43] Peng Y Pei C Zheng Y Wang J Zhang K Zheng Z . A cross-sectional survey of knowledge, attitude and practice associated with COVID-19 among undergraduate students in China. BMC Public Health. (2020) 20:1–8. 10.1186/s12889-020-09392-z 32847554 PMC7447607

[44] Rahman A Sathi NJ. Knowledge, attitude, and preventive practices toward COVID-19 among Bangladeshi internet users. Elect J General Med. (2020) 17:8223. 10.29333/ejgm/8223

[45] Saefi M Fauzi A Kristiana E Adi WC Muchson M Setiawan ME . Survey data of COVID-19-related knowledge, attitude, and practices among Indonesian undergraduate students. Data in Brief. (2020) 51:105855. 10.1016/j.dib.2020.105855 32607405 PMC7291994

[46] Saqlain M Ahmed A Nabi I Gulzar A Naz S Munir MM . Public knowledge and practices regarding coronavirus disease 2019: a cross-sectional survey from Pakistan. Front. Public Health (2021) 9:629015. 10.3389/fpubh.2021.629015 34026708 PMC8133216

[47] Sima R Mariam I Aisha B: Knowledge attitudes and practices (KAP) towards COVID-19: A quick online cross-sectional survey among Tanzanian residents. medRxiv [Preprint]. (2020).

[48] Ssebuufu R Sikakulya F Binezero SM Wasingya L Nganza SK Ibrahim B . Awareness, knowledge, attitude and practice towards measures for prevention of the spread of COVID-19 in the Ugandans: a nationwide online cross-sectional survey. medRxiv [Preprint]. (2020). 10.1101/2020.05.05.20092247 PMC7793670 33425842

[49] Tadesse AW Abebe NM Tadesse SE Wube MC Abate AA. Preventive practice and associated factors towards COVID-19 among college students in Amhara region, Ethiopia: A cross-sectional study. Ethiopian J Health Sci. (2021) 31:3–14. 10.4314/ejhs.v31i1.2 34158747 PMC8188108

[50] Tomar BS Singh P Nathiya D Suman S Raj P Tripathi S . Indian communitys knowledge, attitude, & practice towards COVID-19. medRxiv [Preprint]. (2020). 10.1101/2020.05.05.20092122

[51] Bates BR Moncayo AL Costales JA Herrera-Cespedes CA Grijalva MJ. Knowledge, attitudes, and practices towards COVID-19 among ecuadorians during the outbreak: an online cross-sectional survey. J Comm Health. (2020) 9:1–10. 10.1007/s10900-020-00916-7 32915380 PMC7483492

[52] Ulhaq ZS Kristanti RA Hidayatullah AA Rachma LN Susanti N Aulanni'am A. Data on attitudes, religious perspectives, and practices towards COVID-19 among Indonesian residents: a quick online cross-sectional survey. Data Brief. (2020) 32:106277. 10.1016/j.dib.2020.106277 32984470 PMC7494484

[53] Zhong BL Luo W Li HM Zhang QQ Liu XG Li WT . Knowledge, attitudes, and practices towards COVID-19 among Chinese residents during the rapid rise period of the COVID-19 outbreak: a quick online cross-sectional survey. Int J Biol Sci. (2020) 16:1745. 10.7150/ijbs.45221 32226294 PMC7098034

[54] Alahdal H Basingab F Alotaibi R. An analytical study on the awareness, attitude and practice during the COVID-19 pandemic in Riyadh, Saudi Arabia. J Infect Public Health. (2020) 13:1446–52. 10.1016/j.jiph.2020.06.015 32563674 PMC7832465

[55] Alhazmi A Ali MHM Mohieldin A Aziz F Osman OB Ahmed WA. Knowledge, attitudes and practices among people in Saudi Arabia regarding COVID-19: a cross-sectional study. J Public health Res. (2020) 9:1867. 10.4081/jphr.2020.1867 33042899 PMC7520855

[56] Alobuia WM Dalva-Baird NP Forrester JD Bendavid E Bhattacharya J Kebebew E. Racial disparities in knowledge, attitudes and practices related to COVID-19 in the USA. J Pub Health. (2020) 42:470–8. 10.1093/pubmed/fdaa069 32490519 PMC7313911

[57] Chan EYY Huang Z Lo ESK Hung KKC Wong ELY Wong SYS. Sociodemographic predictors of health risk perception, attitude and behavior practices associated with health-emergency disaster risk management for biological Hazards: the case of COVID-19 pandemic in Hong Kong, SAR China. Int J Environ Res Public Health. (2020) 17:3869. 10.3390/ijerph17113869 32485979 PMC7312582

[58] Dkhar SA Quansar R Saleem SM Khan SMS. Knowledge, attitude, and practices related to COVID-19 pandemic among social media users in J&K, India. Indian J Public Health. (2020) 64(Supplement):S205–10. 10.4103/ijph.IJPH_469_20 32496256

[59] Domiati S Itani M Itani G. Knowledge, attitude, and practice of the lebanese community toward COVID-19. Front Med. (2020) 7:542. 10.3389/fmed.2020.00542 33015096 PMC7461812

[60] Hamza MS Badary OA Elmazar MM. Cross-sectional study on awareness and knowledge of COVID-19 among senior pharmacy students. J Comm Health. (2020) 3:1-8. 10.21203/rs.3.rs-33352/v1 32542552 PMC7295146

[61] Honarvar B Lankarani KB Kharmandar A Shaygani F Zahedroozgar M Rahmanian Haghighi MR . Knowledge, attitudes, risk perceptions, and practices of adults toward COVID-19: a population and field-based study from Iran. Int J Public Health. (2020) 65:731–9. 10.1007/s00038-020-01406-2 32583009 PMC7311321

[62] Hossain MA Jahid MIK Hossain KMA Walton LM Uddin Z Haque MO . Knowledge, attitudes, and fear of COVID-19 during the rapid rise period in Bangladesh. PLoS ONE. (2020) 15:e0239646. 10.1371/journal.pone.0239646 32970769 PMC7514023

[63] Hu Y Zhang G Li Z Yang J Mo L Zhang X . Knowledge, attitudes, and practices related to COVID-19 pandemic among residents in Hubei and Henan Provinces. Nan Fang Yi Ke Da Xue Xue Bao. (2020) 40:733–40. 10.12122/j.issn.1673-4254.2020.05.20 32897216 PMC7277320

[64] Hussain I Majeed A Imran I Ullah M Hashmi FK Saeed H . Knowledge, attitude, and practices toward COVID-19 in primary healthcare providers: a cross-sectional study from three tertiary care hospitals of Peshawar, Pakistan. J Comm Health. (2020) 9:1-9. 10.1007/s10900-020-00879-9 32632645 PMC7338131

[65] Huynh G Nguyen MQ Tran TT Nguyen VT Nguyen TV Do THT . Knowledge, attitude, and practices regarding COVID-19 among chronic illness patients at outpatient departments in Ho Chi Minh City, Vietnam. Risk Manag Healthc Policy. (2020) 13:1571–8. 10.2147/RMHP.S268876 32982515 PMC7500828

[66] Li C Xu J Yue L Shen M Dai M Liu N. Knowledge, attitude, and practice survey regarding coronavirus disease 2019 among residents in Hunan Province. Zhong nan da xue xue bao Yi xue ban = *J Central South Univ Med Sci*. (2020) 45:665–72. 10.11817/j.issn.1672-7347.2020.200277 32879123

[67] Ngwewondo A Nkengazong L Ambe LA Ebogo JT Mba FM Goni HO . Knowledge, attitudes, practices of/towards COVID 19 preventive measures and symptoms: a cross-sectional study during the exponential rise of the outbreak in Cameroon. PLoS Negl Trop Dis. (2020) 14:e0008700. 10.1371/journal.pntd.0008700 32886678 PMC7497983

[68] Nicholas T Mandaah FV Esemu SN Vanessa ABT Gilchrist KTD Vanessa LF . COVID-19 knowledge, attitudes and practices in a conflict affected area of the South West region of Cameroon. Pan Af Med J. (2020) 35:22963. 10.11604/pamj.supp.2020.35.2.22963 33623559 PMC7875723

[69] Olaimat AN Aolymat I Elsahoryi N Shahbaz HM Holley RA. Attitudes, anxiety, and behavioral practices regarding COVID-19 among university students in jordan: a cross-sectional study. Am J Trop Med Hyg. (2020) 103:1177–83. 10.4269/ajtmh.20-0418 32662398 PMC7470553

[70] Pal R Yadav U Grover S Saboo B Verma A Bhadada SK. Knowledge, attitudes and practices towards COVID-19 among young adults with Type 1 Diabetes Mellitus amid the nationwide lockdown in India: a cross-sectional survey. Diabetes Res Clin Pract. (2020) 166:108344. 10.1016/j.diabres.2020.108344 32710997 PMC7375303

[71] Reuben RC Danladi MMA Saleh DA Ejembi PE. Knowledge, attitudes and practices towards COVID-19: an epidemiological survey in North-Central Nigeria. J Comm Health. (2020) 8:1–14. 10.1007/s10900-020-00881-1 32638198 PMC7338341

[72] Salman M Mustafa ZU Asif N Zaidi HA Hussain K Shehzadi N . Knowledge, attitude and preventive practices related to COVID-19: a cross-sectional study in two Pakistani university populations. Drugs Therapy Perspectives: Rational Drug Select Use. (2020) 37:1–7. 10.1007/s40267-020-00737-7 32395069 PMC7210795

[73] Van Nhu H Tuyet-Hanh TT Van NTA Linh TNQ Tien TQ. Knowledge, attitudes, and practices of the Vietnamese as key factors in controlling COVID-19. J Comm Health. (2020) 4:1–7. 10.1007/s10900-020-00919-4 32894387 PMC7476242

[74] Yue S Zhang J Cao M Chen B. Knowledge. Attitudes and practices of COVID-19 among Urban and rural residents in China: a cross-sectional study. J Comm Health. (2020) 7:1-6. 10.1007/s10900-020-00877-x 32757087 PMC7403196

[75] Peng Y Pei C Zheng Y Wang J Zhang K Zheng Z . Knowledge, attitude and practice associated with COVID-19 among university students: a cross-sectional survey in China. Res Square [Preprint]. (2020). 10.21203/rs.3.rs-21185/v1 PMC7447607 32847554

[76] Aynalem YA Akalu TY Gebregiorgis BG Sharew NT Assefa HK Shiferaw WS. Assessment of undergraduate student knowledge, attitude, and practices towards COVID-19 in Debre Berhan University, Ethiopia. PLoS ONE (2021) 16:e0250444. 10.1371/journal.pone.0250444 34003825 PMC8130923

